# Understanding and including ‘pink-collar’ workers in employment-based travel demand models

**DOI:** 10.1371/journal.pone.0301001

**Published:** 2024-04-18

**Authors:** Yiping Yan, Abraham Leung, Matthew Burke, James McBroom

**Affiliations:** 1 Cities Research Institute, Griffith University, Brisbane, Queensland, Australia; 2 School of Engineering and Built Environment, Griffith University, Brisbane, Queensland, Australia; 3 School of Vehicle and Mobility, Tsinghua University, Beijing, China; 4 School of School of Environment and Science, Griffith University, Brisbane, Queensland, Australia; Instituto Politecnico Nacional, MEXICO

## Abstract

The segmentation of commuters into either blue or white-collar workers remains is still common in urban transport models. Internationally, models have started to use more elaborate segmentations, more reflective of changes in labour markets, such as increased female participation. Finding appropriate labour market segmentations for commute trip modelling remains a challenge. This paper harnesses a data-driven approach using unsupervised clustering–applied to *2017–20 South East Queensland Travel Survey* (SEQTS) data. Commuter types are grouped by occupational, industry, and socio-demographic variables (i.e., gender, age, household size, household vehicle ownership and worker skill score). The results show that at a large number of clusters (i.e., k = 8) a highly distinct set of commuter types can be observed. But model run times tend to require a much smaller number of market segments. When only three clusters are formed (k = 3) a market segmentation emerges with one female-dominated type (‘pink collar’), one male-dominated type (‘blue collar’) and one with both genders almost equally involved (‘white collar’). There are nuances as to which workers are included in each segment, and differences in travel behaviours across the three types. ‘Pink collar’ workers are mostly comprised of female clerical and administrative workers, community and personal service workers and sales workers. They have the shortest median commutes for both private motorised and active transport modes. The approach and methods should assist transport planners to derive more accurate and robust market segmentations for use in large urban transport models, and, better predict the value of alternative transport projects and policies for all types of commuters.

## Introduction

The first large strategic travel demand models of the 1950s and 60s, such as the *Chicago Area Transportation Study* in 1959 analysed home-based commute trips by splitting workers into two types: ‘blue-collar’ industrial workers and ‘white-collar’ office workers. Whilst somewhat acceptable in the 1950s, over time this blue collar/white collar duality become less and less representative of the labour force. Economic changes produced greater differentiation in the types of employment offered in increasingly knowledge-based cities. Women began to participate much more in the workforce, and commute, in greater numbers. Travel behaviour studies began to focus on women’s travel from many perspectives, including: gender differences in distances to work; mode of travel; automobile occupancy; and, the propensity to combine multiple destinations in one trip [1–4]. Gender was soon recognised as one of the key socio-demographic influences on commuting behaviour. Women and men who worked in the same occupational category often had different commuting patterns [5]. Nevertheless, many transport models for cities/regions, including Australian transport models, tended to subsume women’s travel by retaining a simplistic blue/white collar market segmentation [6–11]. Cities such as London [12,13] and Paris [14–16] in Europe, and many regional models in North America [17–19] have similar blue/white collar market segmentations in their transport models. But more nuanced and differentiated market segmentations are being developed. This paper reports on a novel typology of workers developed for South-East Queensland, Australia, that can help direct this field towards improved market segmentations that better represent the structure of today’s workforce. Instead of the ‘traditional approach’ of experts deductively searching through travel survey data to identify preferred segmentation of commuters, a data-driven clustering approach was used to explore what different segments emerge when one allows only three clusters, or as many as eight clusters, from the same dataset. As will be shown, this unsupervised clustering analysis produced a more nuanced understanding of workers’ occupational and socio-demographical characteristics. These understanding should help modellers better represent commuting by all workers, including women.

## Background

### Evolution of the labour force

During much of the twentieth century, employment was viewed as full-time and permanent waged, where a male was the main income earner of a (hetero-normative) household and a female was the main domestic carer [20]. Since the latter part of the Twentieth Century, journey-to-work patterns have transformed due to changes in the nature of employment, transportation costs, economic shifts, increasing female workforce participation and public policies that increasingly favoured labour mobility and uncertainty [21,22]. The rise of the ‘gig economy’ and telecommuting workers [23] the expansion of the service industry, and a decline in manufacturing in the West, all added more complexity. Strategic transport models were often only partially updated, usually in piece-meal fashion, to represent the commuter behaviours that emerged.

The term ‘blue collar’ to identify manual labouring workers first appeared at the beginning of the 20th century; it differentiates from the ‘white collar’ that identifies a class of administrative workers [24]. The early mid-century modelling pioneers harnessed these two employment types, and tried to forecast commuting flows for these workers, to help predict peak-hour travel flows, and, in turn, how much road and public transport infrastructure was needed. They did so noting the mode choices of these workers, their travel times, and their trip movements, withing commuter’s observed spatial and temporal constraints. This developed these understandings from the first household travel surveys used to develop and calibrate these models. In this immediate post-World War II period, economies in the US, UK and Australia were dominated by manufacturing, mining and industry [25]. With high tariffs on imported goods, firms hired workers in steelworks, automobile and chemical plants, and a range of other factories. With low female workforce participation, and with limited computing power, a simple white/blue collar split was a reasonable approach for transport modellers to employ.

A series of structural changes in the 1970s and 1980s saw a shift towards a post-industrial future. Reaganomics in the USA, Thatcherism in the UK and the Hawke/Keating reforms in Australia all saw tariffs reduced, state utilities such as airlines and airports sold off, and the economy increasingly liberalised. Western nations began to import more manufactured goods than they made locally. Machines replaced many workers in the factories and then in the mines. Knowledge work, including in finance, banking and education, and services work, including in health, began to increase as the total number of employees in manufacturing and labouring fell. At the same time, women’s liberation movements achieved increasing equality in access to work, and in pay rates. Discriminatory practices were outlawed and/or discouraged. Childcare services expanded. Many households obtained two or more cars. It became a different world.

The workers that today form the ‘blue-collar’ and ‘white collar’ market segments of the labour force differ from those of previous eras. Workers in many Australian or US factories may have more advanced skills and higher education training than the workers of the past. Today’s workers may have many different employers over a lifetime and be increasingly flexible in terms of their work arrangements. The previously unionised tradesmen (electricians, plumbers, etc.) who worked for big construction firms or utilities in the 1960s are today mostly self-employed contractors, effectively small business owners whose key assets are their labour and their skills. Within the ‘white-collar’ segment we now see ‘portfolio workers’, who trade on their knowledge and may work as contractors to multiple employers, perhaps based from a co-work space (shared by multiple micro-firms rather than one big employer) in the inner city. The ‘gig-economy’ of smart-phone apps (Uber, Deliveroo, etc.) has created a new class of ‘platform’ worker, with minimal labour protections [26]. To classify all these worker types using the 1960s division of ‘blue collar’ and ‘white collar’ doesn’t seem appropriate. But again, there has been little analysis of these trends, or of what a more robust market segmentation might look like for commuter travel demand modelling.

To focus on labour market segmentation alone is not to ignore other changes for these workers. Spatially, middle- and upper-income workers have reclaimed once industrial inner-city areas, including waterfronts, pushing lower-income workers to the city fringe. Inner-city workers in a particular profession can have very different attitudes and perceptions to their outer-suburban peers. Inner-city gentrifiers tend to express more environmentally and socially progressive attitudes and a preference for urban lifestyles and amenities, which suggests that they may engage in more sustainability-conscious behaviours, including in travel [27–30]. The rise of inner-city cycling (with Washington D.C. going from 1% to 5% cycling rates in the last decade) is partly seen as a result of in-migration by knowledge workers. Numerous studies have indicated that residential context, socio-economic characteristics like employment type, and environmental attitudes are likely to affect commuters’ behaviour [31]. It is not the main intention of this paper to study such socio-demographic changes in-depth; these are future research agendas. But one needs ways to keep transport models representative of cities and their citizens.

### Employment market segmentation in transport models

The 1955 *Detroit Metropolitan Area Traffic Study* [32] crudely took ratios of workers in industrial plants per 1,000 residents, and ratios of workers located within the core area of the central business district per 1,000 residents to represent ‘blue’ and ‘white’ collar workers (albeit without actually using those terms). The field advanced and by 1965 the *Brisbane Transportation Study* adopted a more detailed classification of the employment market, by grouping such industries as primary production, manufacturing, building and public services, business services and commerce, public authorities and professional services, personal services and other industries [33]. Despite all the changes in labour force markets during the last 60 or more years, the *Brisbane Strategic Transport Model* (BSTM), the successor of that work, is still using ‘blue/white collar’ market segmentation [10]. Larger cities with more complex commuter markets have moved on, with Sydney’s model today including ‘pink-collar’ workers to represent female-dominated service jobs, and ‘gold-collar’ workers to represent advanced business and finance industry jobs [34].

The use of deductive approaches to identify and analyse a ‘pink-collar’ market segment in household travel surveys, and include this in transport modelling, is becoming more common, for good reason. There are many constraints on women’s travel, often due to childcare and other family care responsibilities, and due to different motor vehicle availability. This limits their work trip distances and commuting mode preferences [35,36]. Nevertheless, studies about how to incorporate these gender differences in mode choice models and other parts of the large strategic transport models, are rarer. Given the heavy dominance of men in the transport planning/engineering field, the potential for unconscious male-bias when using deductive approaches is probably high. That is, male modellers are not deliberately choosing sub-optimal classifications when searching for and analysing commuters in female-dominated professions. It’s that they may unconsciously bring biases to their choices and accidentally err. It’s relatively easy to assume female workers in a particular occupation will have similar commuting behaviours as men, when they do not; or vice versa. For this reason, inductive data approaches are generally preferable for such tasks, as they remove or reduce the potential for researcher biases.

## Approach and methods

### Data collection

This paper uses data from the *South East Queensland Travel Survey 2017–2020* (SEQTS), made publicly available in an open data portal by the Queensland Department of Transport and Main Roads (DTMR) [37]. The SEQTS data used a stratified, multi-stage clustered sampling technique. 101,616 trips were recorded from 36,264 respondents living in 14,715 households, with a response rate of about 50%. Travel diaries were completed for all members of the household aged five and over, capturing information on all trip stages for each trip. More information on the data collection process is available from DTMR [37].

**Fig 1:**
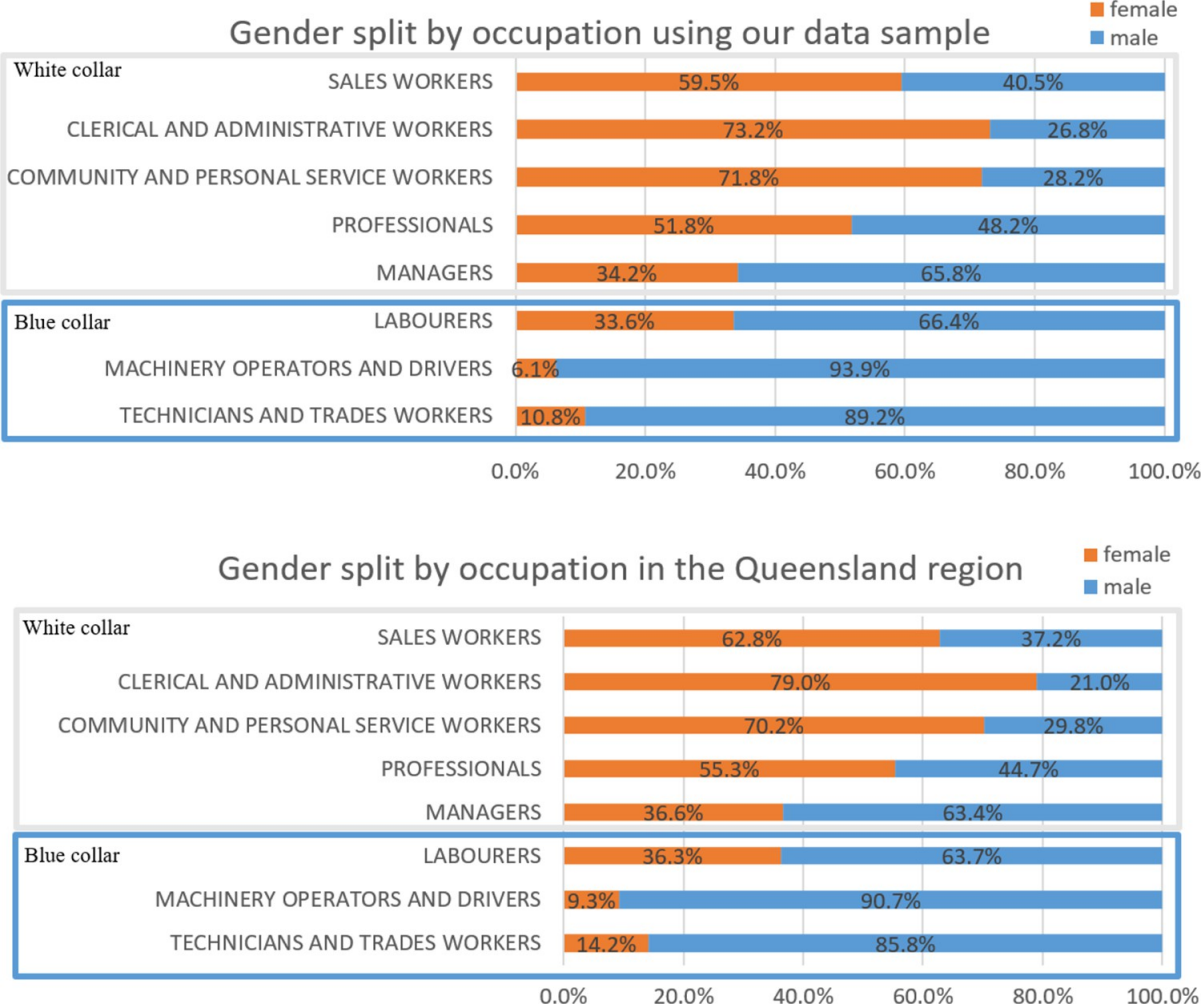
Gender split by occupations (our data sample vs. ABS data in the region).

A ‘main mode’ was allocated to each trip by the DTMR, including journey-to-work trips. The ‘main mode’ is the mode used on any trip stage in an overall trip, using a hierarchy (from high to low) of public transport modes, then private vehicle travel, then cycling, then walking. If a trip is made by both car and public transport it is categorised as public transport, and so on. The sample included 9,150 workers’ (56% males and 44% females) with journey-to-work trip records, with five cases excluded due to abnormally large commutes (>200km that indicate travelling outside the region). Percentages of employed persons by occupation in our sample, and in the region’s comparable census data, are provided in Table 2. The comparison between gender split by occupation in our sample and in the census data are shown in Fig 1. As presented below, the dataset adequately reflects the actual commuters of the region, albeit with some under- and over-representation of women in a few categories in the SEQTS data sample. The dataset we adopted was collected before the Covid-19 pandemic, and is not ‘polluted’ by the forced telecommuting seen in the region for much of 2020–21.

### Selection of parameters in the market segmentation model

Standard occupation classifications provided in census data have been commonly used by transport modellers. The underlying assumption of market segmentation is that people with different characteristics place different importance on different aspects of service [38,39]. Employees’ occupational and socio-demographic characteristics should play a significant role in impacting their commuting behaviour. In Australia the *Australian and New Zealand Standard Classification of Occupations* (ANZSCO 2013, Version 1.2) has 1,023 listed occupations classified into 8 major groups: managers, professionals, technicians and trade workers, community and personal service workers, clerical and administrative workers, sales workers, machinery operators and drivers, and labourers [40], as shown in Table 1.

**Table 1:** Percentage of different occupations in our data sample vs. ABS census data for the region.

Occupation type	Sample size	Percentage by occupation	
		(Sample data, 2017–19)	(ABS Census data, 2016)
Managers	1,247	13.5%	12.0%
Professionals	2,313	25.0%	21.7%
Community and Personal Service Workers	955	10.3%	11.2%
Clerical and Administrative Workers	1,212	13.1%	14.3%
Sales Workers	650	7.0%	10.0%
Technicians and Trades Workers	1,505	16.2%	13.6%
Machinery Operators And Drivers	555	6.0%	5.9%
Labourers	712	7.7%	9.7%
Miscellaneous	118	1.3%	1.6%

As shown in Table 2, ANZSCO Skill Level is a skill-based classification used to classify all occupations and jobs, and group them into successively broader categories for statistical and other types of analysis based on the similarity of their attributes in the Australian and New Zealand labour markets. Using aspects of both skill level and skill specialisation, ‘sub-major’ groups are then collected into eight ‘major’ groups. ANZSCO1 (Level One) represents the broadest level of ANZSCO with 8 major groups and ANZSCO3 (Level Three) represents for minor groups subdivided from higher level groups. ANZSCO classifies occupations according to two criteria–skill level and skill specialisation. In this study, the skill specialisation for ANZSCO3 is adopted as it is more comprehensive. Skill Levels, ranging from 1 (highest) to 5 (lowest), is measured by the level of formal education and training, previous experience, and/or on-the-job training, required to competently perform in that occupation. In the current BSTM, the major groups are crudely aggregated into two main types to form the ‘white/blue collar’ market segmentations used in the mode choice model (see Table 1).

**Table 2:** Definition of blue/white collar by ANZSCO Level 1 major groups.

ANZSCO Level 1 (Major Groups)	‘Collars’ in BSTM	ANZSCO Level 2 (Sub-Major Groups)	ANZSCO Level 3 (Minor Groups)	ANZSCO Level 4 (Unit Groups)	ANZSCO Level 5 (Occupations)
1. Managers	White	4	11	38	99
2. Professionals	White	7	23	100	318
3. Technicians and Trades Workers	Blue	7	21	66	179
4. Community and Personal Service	White	5	9	36	105
5. Clerical and Administrative Workers	White	7	12	33	80
6. Sales Workers	White	3	5	19	37
7. Machinery Operators and Drivers	Blue	4	7	22	77
8. Labourers	Blue	6	9	44	128
Total Number of classifications: (8)	(2)	(43)	(97)	(358)	(1023)

Unsupervised clustering analysis is an alternative approach to use such data to generate an alternative market segmentation. In essence, it reveals subgroups within heterogeneous data, where each individual cluster has greater homogeneity than the whole [41]. The methods selected for this work consisted of three major stages: 1) data preparation, including feature selection, and extraction; 2) use of an unsupervised PAM (partition around medoids) clustering algorithm to analyse datasets made of mixed-typed data; and, 3) comparing results for different numbers of clusters (k = 2, 3, 4, 5, 6, 7, 8) to help identify an optimal k-value using an average silhouette width measure [42].

The selection of variables for the cluster analysis was based on data availability within the SEQTS and guided by prior research in the field [43–45]. Variables that were too similar were selectively eliminated to avoid co-linearity. The socio-demographic characteristics finally included were: gender, age, occupation groups (ANZSCO Classification of Occupations), Skill Level, industry type, household size, household vehicle ownership, and household bicycle ownership. This approach did omit some variables known to influence travel behaviour. The SEQTS itself does not have any attitudinal data, such as environmental attitudes or attachment to particular modes like driving. This is a major limitation common to most other HTS datasets around the world.

### Unsupervised Partitioning Around Medoids (PAM) clustering

Unsupervised methods have an advantage over traditional clustering methods (such as hierarchical) as they do not require prior knowledge and are less subjective. Also, compared to the more commonly used k-means clustering algorithm, Partitioning Around Medoids (PAM) is more intuitive and robust to noise and outliers in the underlying data, due to the properties of distances being used. PAM is also capable of analysing mixed-type data, where numeric, nominal, or ordinal features coexist. The main disadvantage of PAM is its unsuitability for clustering non-spherical (arbitrary shaped) groups of objects, but this became irrelevant as the clustering formed relatively spherical shapes.

Increasing the number of clusters raises the risk of overfitting, by definition. We limited our analysis to a maximum of eight clusters (k = 2 to 8) and calculated the silhouette coefficients for each k to qualify the relevancy of the chosen number of clusters from a statistical perspective. In distance-based clustering of mixed-type data, a good performance of detecting clusters can be achieved using Gower’s dissimilarity followed by PAM [46,47]. The Gower distance metric was used to measure proximity or similarity across individuals within our dataset. Most simply, Gower distance is computed as the average of partial dissimilarities across individuals, in which each numeric-valued feature is standardised, and the distance is calculated as the average of all feature-specific distances. However, if variables are of mixed (qualitative as well as numeric) types, partial dissimilarity is calculated differently. For numeric features, it is computed as the ratio between absolute difference of observations X_i_ and X_j_ and maximum range observed from all individuals (thus scaling all dissimilarities to lie between 0 for identical, and 1 for maximally dissimilar):

$$d_{ij}^{\left(f\right)}=\frac{\left|x_{i}-x_{j}\right|}{\left|x_{MAX}-x_{Min}\right|}$$

The Gower distance’s formula is provided below with n representing the sample size:

$$d(i,j)=\frac{1}{n}\sum_{k=1}^{n}d_{ij}^{\left(f\right)}$$

As a classical partitioning technique of clustering, a K-medoid algorithm is applied to cluster the dataset into k clusters fixed a priori, incorporating the Gower distance metrics result. Silhouette coefficient is used, and a high value of that index implies well clustered groups [48]. In this study, Silhouette width particularly measures whether workers that have similar socio-economic traits and occupation type to each other are placed in the same cluster and whether clusters are tightly bound with a substantial distance from each other.

**Fig 2:**
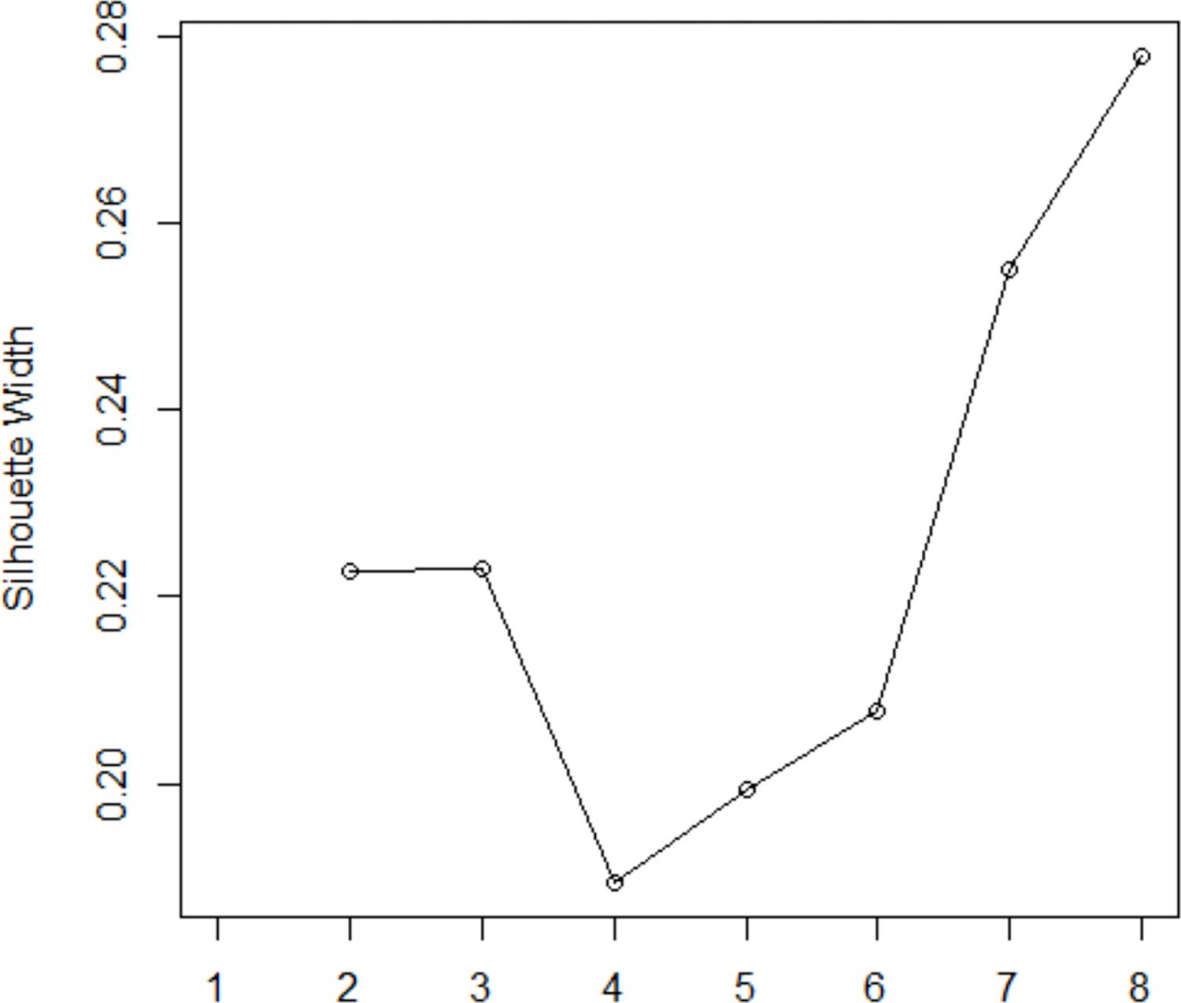
Silhouette width results when clusters size ranging from 2 to 8.

In Fig 2, when k = 4, the data points are not compact within the cluster to which they belong and there is more overlapping between clusters; this is sub-optimal. It seems that increasing k value to 8 only insignificantly improve the width result from k being 2 or 3. Considering the result from average Silhouette width measure, as well as the significant overall model run-time savings of having fewer market segments in strategic transport models, the most useful number of clusters for the SEQTS dataset was just three.

All the clustering analysis was performed using *R* software. Once market segments were identified, the travel behaviour of these groups was then explored using *Python* with the same SEQTS dataset. The variables calculated included: mode shares; and, trip distances by mode, with the latter represented as violin plots for ease of interpretation.

## Results

### Occupational clusters when k = 8 and when k = 3

Tables 3 and 4 show the occupational clusters revealed when there were eight clusters (k = 8) and when there were only three (k = 3), respectively. These serve difference purposes, as the k = 8 solution is more useful for refined models. As the number of clusters reduces, one can see the merging of these groups in interesting ways, and how the unstructured classification process shifts commuters into different groups when forced to place them in a limited number of bins (i.e., the transition from k = 8 to k = 3).

**Table 3:** Summary results at highest level of disaggregation (k = 8).

Cluster No.	1	2	3	4	5	6	7	8
Dominant Age Group	10 (45–49 years)	10 (45–49 years)	10 (45–49 years)	10 (45–49 years)	9 (40–44 years)	9 (40–44 years)	10 (45–49 years)	8 (35–39 years)
Dominant Sex (Male/Female)	Female (931/1017)	Female (1282/1360)	Male (735/755)	Male (841/998)	Male (1453/1536)	Male (1166/1166)	Female (1373/1373)	Male (769/944)
Dominant ANZSCO1 (count)	Community and professional service workers (755)	Clerical and administrative workers (869)	Machinery operators and drivers (532)	Managers (964)	Technicians and trades workers (1425)	Professionals (1114)	Professionals (1199)	Labourers (626)
Dominant Industry (count)	Health (490)	Retail (453)	Transport (405)	Construction (216)	Construction (582)	Education (226)	Education (475)	Other (347)
Household size (median/mean)	3/3.016	2/2.740	3/3.057	3/3.068	3/3.083	3/3.068	2/2.805	3/3.059
Household vehicle number (median/mean)	2/2.293	2/2.242	2/2.291	2/2.354	2/2.345	2/2.148	2/2.183	2/2.144
Bike ownership(median/mean)	1/1.439	1/1.239	1/1.334	2/1.934	1/1.553	2/1.916	1/1.535	1/1.314
Workplace is in Central Business District (CBD) (mean)	3.8%	11.8%	3.8%	11.9%	4.95%	19.5%	11.5%	5.6%
Skillscore (median/mean)	4.0/3.641	3.5/3.663	4.0/4.054	1.50/1.434	3.0/2.884	1.0/1.096	1.0/1.10	4.5/4.603
Mode share of active transport*	3.44%	1.7%	0.7%	1.4%	1.6%	5.6%	2.7%	3.4%
Mode share of private motorised vehicle	88.4%	84.1%	94.2%	88.5%	93.9%	76.2%	83.2%	89.8%
Mode share of public transport	7.77%	13.7%	4.6%	9.9%	4.5%	18.0%	13.7%	6.5%
Median trip distance (km) of active transport	0.93	1.23	2.11	5.90	1.94	4.54	1.54	1.285
Median trip distance (km) of private motorised vehicle	10.97	13.115	19.53	17.72	18.795	16.005	13.05	14.255
Median trip distance(km) of public transport**	20.61	22.79	18.67	24.45	26.62	19.335	18.71	20.490

*Active transport includes journeys made by walking only, or by cycling, but does not include public transport trips where these modes were used for the first or last mile.

**Median public transport trips being longer than the private car trips was unexpected. However, Brisbane’s express busway and rail networks carry many suburban commuters into the centre; there are many inter-city commute trips made, including between the Gold Coast, Ipswich, the Sunshine Coast and Brisbane; there are almost no cross-suburban public transport routes, requiring transfers in Brisbane’s CBD; and, the city’s road networks tend to offer more direct routes between Origin-Destination pairs, and lesser trip distances, than the rail corridors.

**Table 4:** Summary results at three levels of disaggregation (k = 3).

Cluster	1 (Blue Collar)	2 (Pink Collar)	3 (White Collar)
Dominant Age Group	9 (40–44 years)	9 (40–44 years)	10 (45–49 years)
Dominant Sex (Male/Female)	Male (3732/3801)	Female (2060/2391)	Female (1892/2957)
Dominant ANZSCO1 (count)	Technicians and trades workers(1408/3801)	Clerical and administrative workers (1049/2391)	Professionals (2231/2957)
Dominant Industry (count)	Construction (1090/3801)	Retail (605/2391)	Health (928/2957)
Household size (median/mean)	3/3.1	3/2.9	3/2.9
Household vehicle number (median/mean)	2/2.3	2/2.3	2/2.2
Bike ownership (median/mean)	1/1.7	0/1.2	1/1.6
Skillscore (median/mean)	3/3.0	4/3.9	1/1.3
Workplace is in Central Business District (CBD) (mean)	6%	9%	14%
Mode share of active transport	63/3793 (1.7%)	61/2379 (2.6%)	111/2947 (3.8%)
Mode share of private motorised vehicle	3512/3793 (92.6%)	2045/2379 (86.0%)	2400/2947 (81.4%)
Mode share of public transport	218/3793 (5.7%)	273/2379 (11.5%)	436/2947 (14.8%)
Median trip distance(km) of active transport	2.08	1.18	2.89
Median trip distance(km) of private motorised vehicle	18.405	12.28	13.75
Median trip distance(km) of public transport	25.765	21.07	19.225

Interrogating these results one can see that at k = 8, the disaggregated commuter types include the more traditional ‘blue collar’ (Groups 3, 5 and 8), and ‘white collar’ (Groups 4, 6, 7) groupings. But there are also two other distinct female-dominated clusters: community and professional service workers (Group 1), and clerical/administrative workers mostly across the retail and health industries (Group 2). Neither of these clusters fits either the ‘white’ or ‘blue’ collar categories. Interestingly, when aggregated to just six clusters (k = 6, not shown in this paper), these mostly female community and personal service workers, and clerical and administrative workers stay almost the same. When further aggregated to only four clusters (k = 4, not shown in this paper), the two disaggregated female-dominated clusters merge into one single cluster.

When forced into just three clusters (k = 3), the male-dominated professional workers group and the female-dominated professional workers group seen in Table 2 seem to merge into one cluster, the ‘white-collar’ group 3 in Table 4. 64% of its members are female. There is also female-dominated cluster (86.2% female) which we refer to as ‘pink-collar’. Third, there is a ‘blue-collar’ cluster, 98.2% of whom are male. Details about the median/average value of socio-demographic characteristics and mode share/median trip distance by mode of all these groups (when k = 8 and 3) are summarised in Tables 3 and 4, respectively.

**Fig 3:**
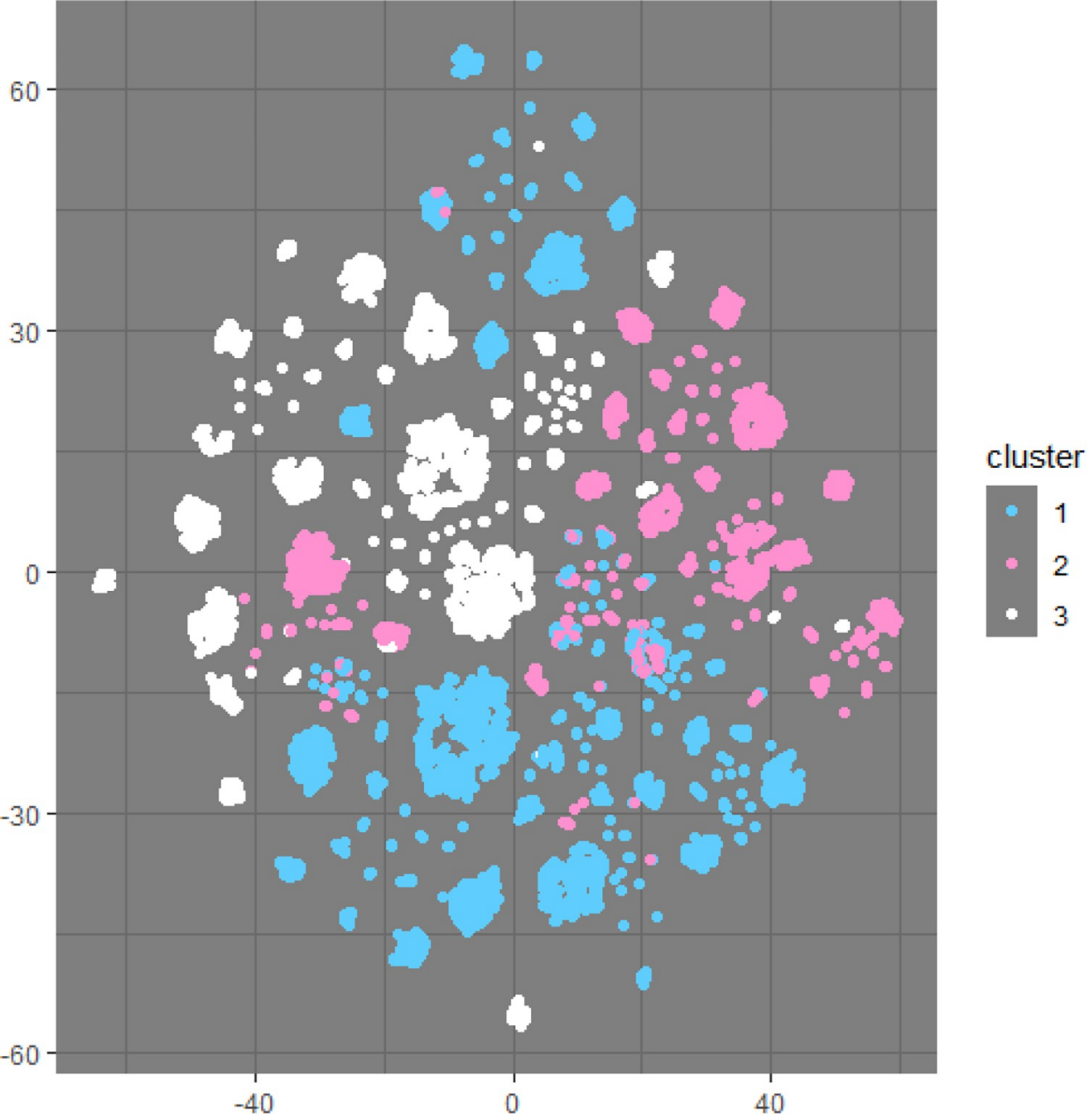
The t-SNE representation for three clusters.

The t-Distributed Stochastic Neighbour Embedding (t-SNE) method is a specialised technique for reducing dimensionality, making it especially effective for visualising high-dimensional data within a low-dimensional space (e.g., two- or three-dimensional) while preserving local structure [49]. Using t-SNE, the data can be visualised into a two-dimensional plot (Fig 3) showing the clusters of the blue, white and pink collar k = 3 solution as presented in Table 4. It should be noted that this is visualisation technique to tell how far apart the clusters are in a low-dimensional, they not meant for quantitative interpretation.

### Profile of the three types of commuters

**Fig 4:**
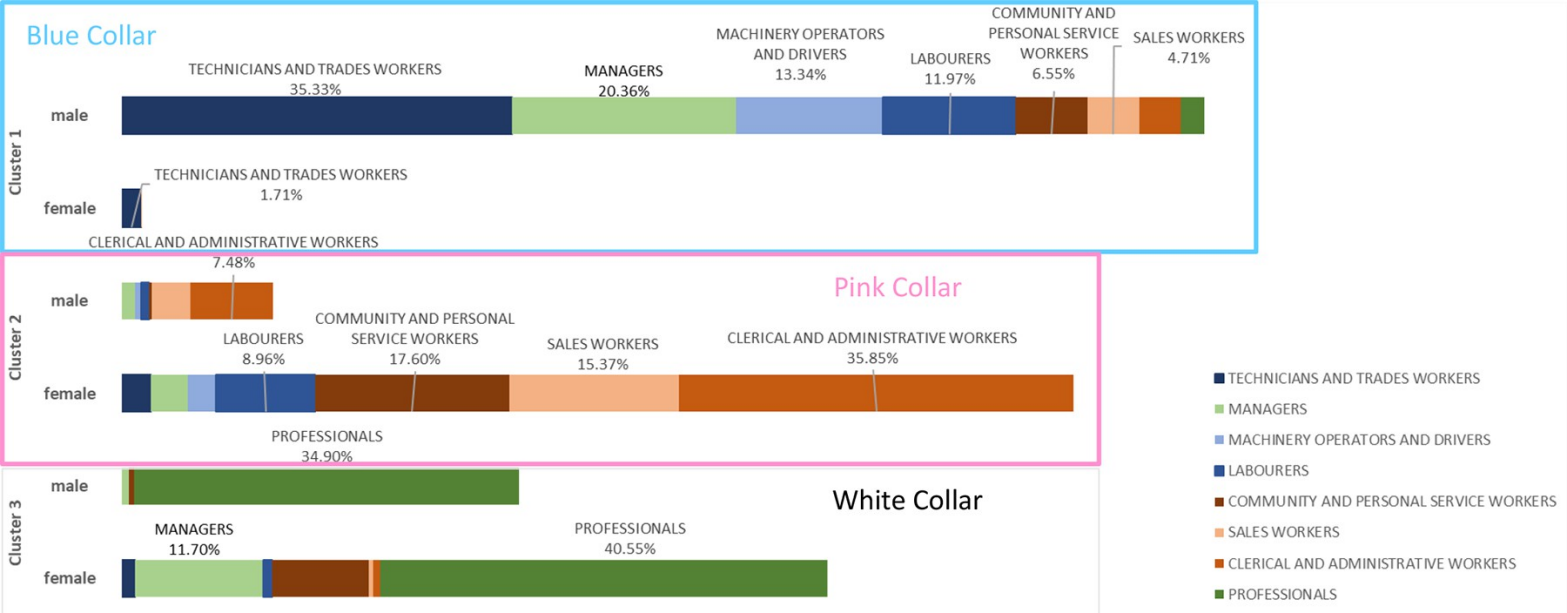
Component analysis of three clusters by gender and occupation.

Fig 4 provides further occupational breakdown of the three clusters (k = 3). The ‘pink-collar’ group is mainly comprised of female clerical and administrative workers (35.85%), community and personal service workers (17.6%) and sales workers (15.37%) with a 3.9 average skill score. The other two groups closely fit the conventional ‘white-collar’ and ‘blue-collar’ grouping. Managers and Professionals account for more than 85% of the ‘white-collar’ workers, sharing a 1.3 average skill score. 98% of ‘blue-collar’ workers are male, mostly working as technicians and trades workers, managers, and machinery operators and drivers, and as laborers.

**Fig 5:**
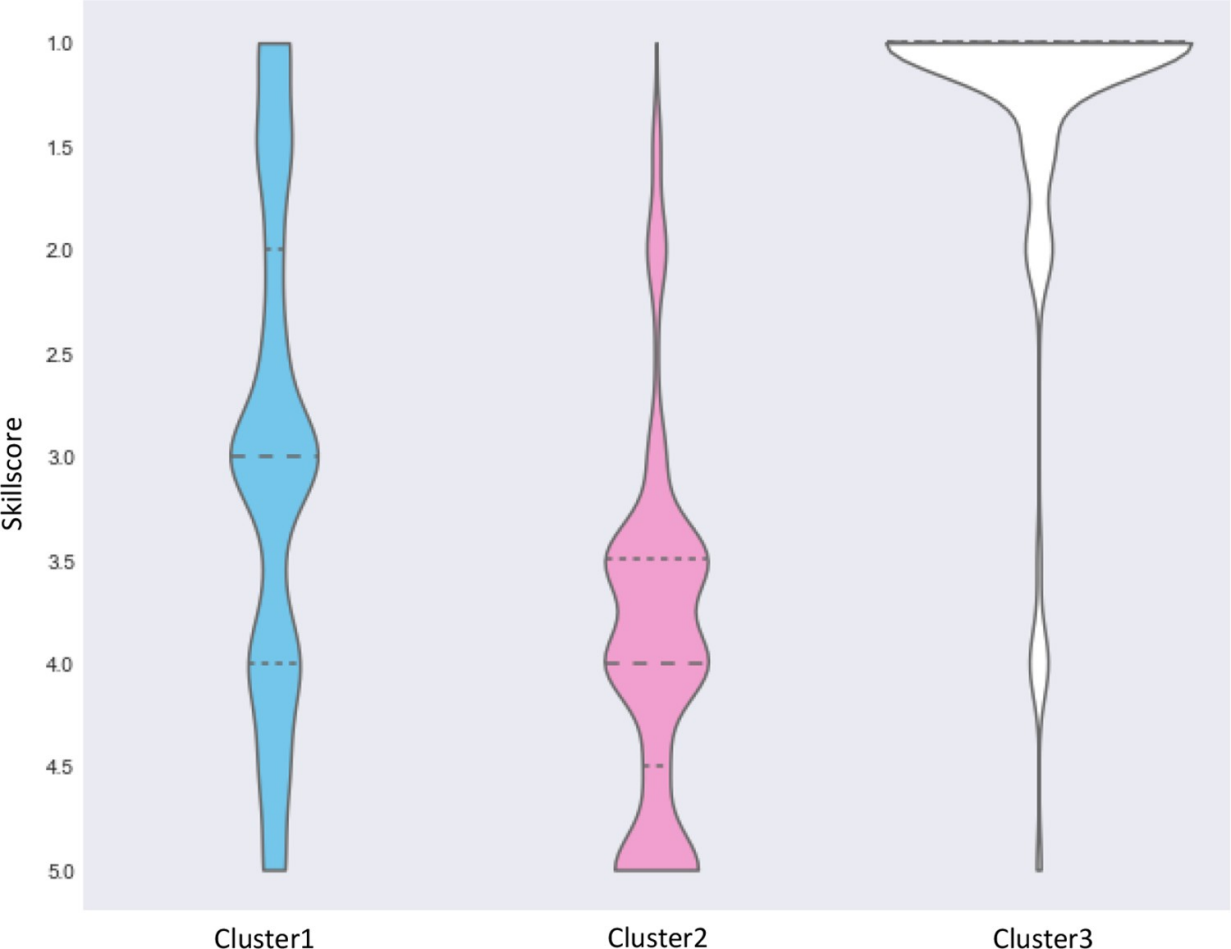
Distribution of Skill Score across the three clusters.

Fig 5 suggests a noticeable difference in the distribution of Skill Score across the three clusters. Most ‘white-collar’ workers have higher levels of knowledge or formal training. ‘Pink-collar’ workers have lower levels. The skill level for blue-collar workers appears to be more evenly distributed, reflecting the increased skills and training required of many workers in these industries today. Locations of employment also differed by cluster. More ‘white-collar’ workers worked in the central business district of Brisbane; ‘pink-’ and ‘blue-collar’ workers’ worked more in suburban areas.

### Travel behaviour analysis of three types of workers

**Fig 6:**
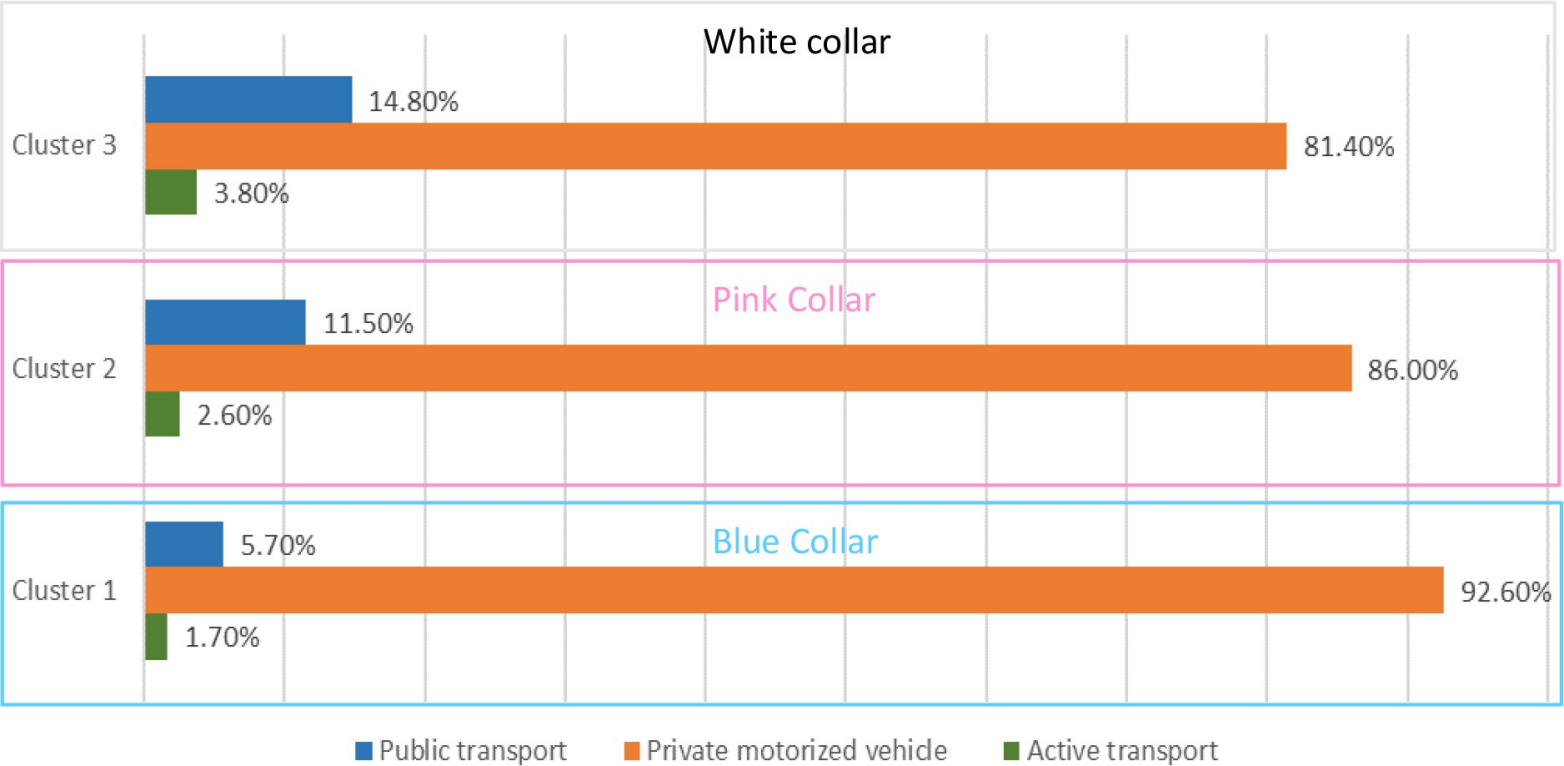
Mode share comparison across the three clusters.

**Fig 7:**
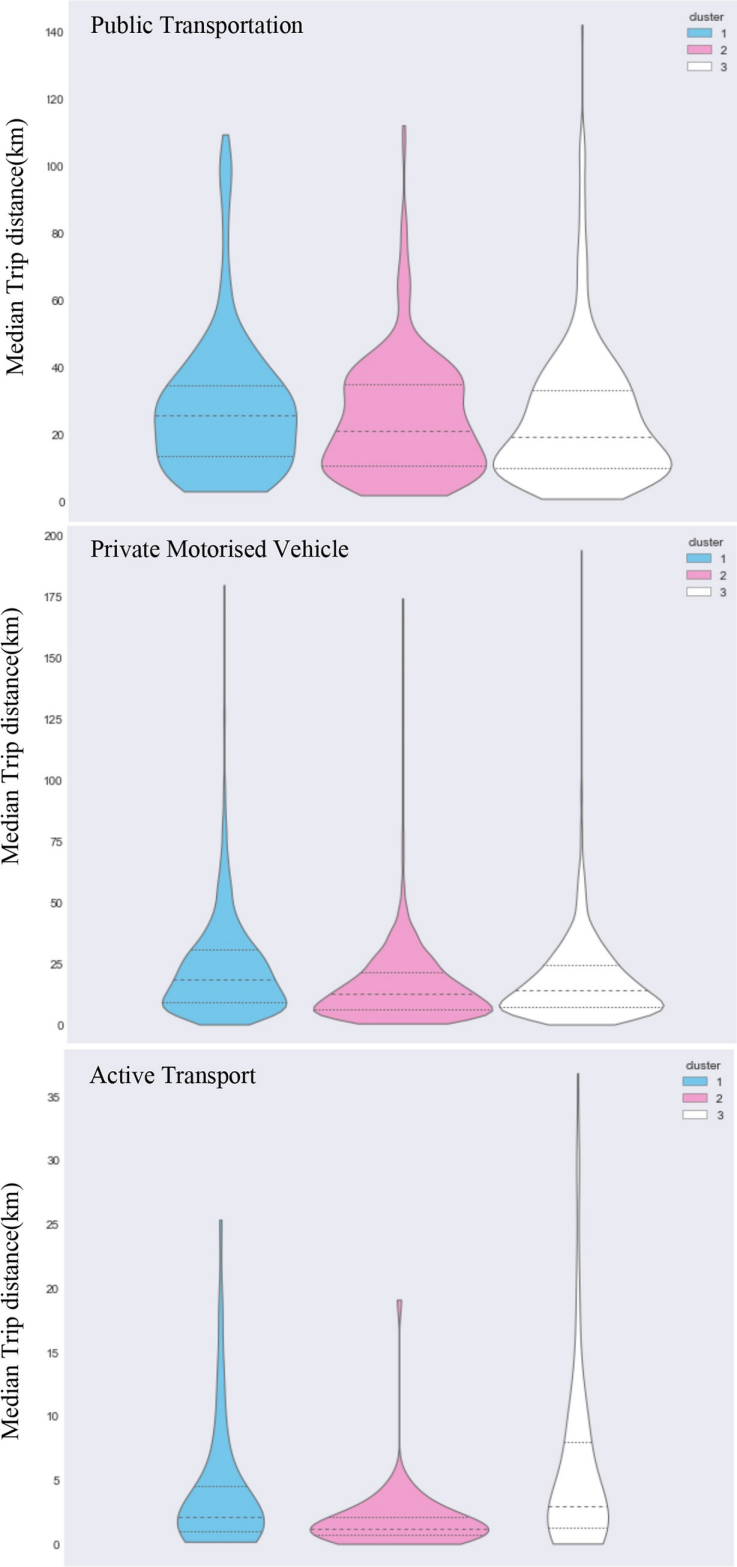
Median trip distance distribution by mode for the three clusters.

Figs 6 and 7 shows commuting behaviours of the three clusters based on respondents’ journey-to-work trips only. Private motor vehicle dominated commuting across the three groups: 81.4% of all commutes made by ‘white-collar’ workers; 86% for ‘pink-collar’ workers, and 92.6% for ‘blue-collar’ workers. In terms of median trip distances by private motor vehicle, the white-collar workers tended to commute shorter distances (13.7 km) than blue-collar workers (18.4 km) whilst pink-collar workers have the shortest average driving distance (12.3 km). The proportion of white-collar workers commuting by public transportation was almost three times greater than the blue-collar workers (14.8% versus 5.7%). However, blue-collar workers tended to travel longer distances when commuting by public transport when compared to white-collar workers (25.8 km vs 19.2 km, respectively). Pink-collar workers have a modest share of commutes made by public transport (11.5%) but have a similar median trip distance for these trips to ‘white collar’ workers (21.1 km vs. 19.2km, respectively). White-collar workers were mostly likely to use active transport modes, and their trip distances were almost twice as far as the ‘pink-collar’ group; this would have health benefit implications for the different groups [50,51]. These results are consistent with previous research on Australian households, showing that women often take work closer to home in the suburbs to be closer to schooling and childcare [52]. The results suggest that commuting behaviours between the three clusters are statistically dissimilar.

## Discussion

There is a need to continue to improve the accuracy of city-region transport models, to generate more robust demand forecasting. This paper has made a number of small contributions towards this task; the analysis being just one modest step in identifying potential improvements. Firstly, the results confirm that those who deductively identified and included pink-collar market segments in urban transport model elsewhere were on the right track. Second, the paper has shown that inductive clustering techniques can be used to explore commute types at a number of levels and see what is happening as different groupings are aggregated. This approach is not just theoretically more robust, the analysis shows it can directly improve market segmentation choices. The approach also allows each city to go and explore its own datasets and come up with reasonable and justifiable ‘solutions’ for the market segmentation problem, regardless of its peculiar employment market. What may work for New York may be very different to that of Brisbane, and different again in Honolulu. As household travel survey data are increasingly made available freely via open data portals, the methods developed in this paper should help planners, modellers and researchers across a litany of cities improve the accuracy of their models.

There are limitations and one must be cautious in interpreting the results. Female-dominated or male-dominated clusters, and their travel behaviour, should not be conflated with the commuting behaviour of men or women, per se. There is nuance needed in taking these results through and operationalising them in mode choice models. The results suggest that the conventional blue/white collar segmentation strategy for transport models may fail to sufficiently represent female workers’ travel behaviour appropriately, at least in Australia. However, this conjecture should be tested by comparing the predictive ability, by gender, of models using a blue/white collar segmentation to that of models using the blue/white/pink collar segments identified in this paper.

There are also other data-driven approaches that could take this work further. Latent class mode choice studies could provide a richer picture and additional improvements. There also appears significant worth in further exploring the travel characteristics of the two distinct female-dominated clusters of commuters’ travel identified in Table 3 when there were eight clusters (k = 8). This suggests that pink-collar workers are potentially two key groups (we label these, ‘*aqua-collar*’ and ‘*purple-collar*’ commuters?). When computing power and transport modelling advances to the point where a higher level of disaggregation is possible without pushing out model run times, it may be of value to include such differentiation in the market segmentation problem. At the present time, however, the benefits of doing so in model accuracy are likely out-weighed by the problems of model complexity and run-time. Finally, methodological and cultural change will continue in the future. Transport researchers may look back at this paper and admonish it for omitting a litany of new variables, and for its own conscious and unconscious decisions. That is to be both expected, and encouraged.

## References

[1] McGuckin N, Zmud J, Nakamoto Y. Trip-Chaining Trends in the United States: Understanding Travel Behavior for Policy Making. Transportation Research Record. 2005;1917: 199–204. doi: 10.1177/0361198105191700122

[2] Gordon P, Kumar A, Richardson HW. Gender Differences in Metropolitan Travel Behaviour. Regional Studies. 1989;23: 499–510. doi: 10.1080/00343408912331345672

[3] Nosal Hoy K, Puławska-Obiedowska S. The Travel Behaviour of Polish Women and Adaptation of Transport Systems to Their Needs. Sustainability. 2021;13: 2693. doi: 10.3390/su13052693

[4] Pickup L. Women’s Gender-Role and its Influence on Travel Behaviour. Built Environment (1978-). 1984;10: 61–68.

[5] Rosenbloom S, Burns E. Why Working Women Drive Alone: Implications for Travel Reduction Programs. UC Berkeley: University of California Transportation Center; 1994. Available: https://escholarship.org/uc/item/4x17v3f1.

[6] Infrastructure Victoria. Victorian Land Use and Transport Integration (VLUTI) Model Architecture Report. Government of Victoria; 2021. Available: https://www.infrastructurevictoria.com.au/wp-content/uploads/2021/08/Victorian-Land-Use-And-Transport-Integration-VLUTI-Model-Architecture-Report.pdf.

[7] Olaru D, Taplin J, Taylor M, Biermann S. Independent Transport Modelling Review: Final Report. 2014. Available: https://research-repository.uwa.edu.au/en/publications/transport-modelling-review-independent-review.

[8] Roads and Maritime Services of New South Wales. Traffic Modelling Guidelines. 2013. Available: https://roads-waterways.transport.nsw.gov.au/business-industry/partners-suppliers/documents/technical-manuals/modellingguidelines.pdf.

[9] Morris J, Roddis S, Spiridonos F. Women’s changing role: implications for the transport task and for modelling personal travel patterns. Canberra, ACT, Australia; 2010. Available: https://australasiantransportresearchforum.org.au/wp-content/uploads/2022/03/2010_Morris_Roddis_Spiridonos.pdf.

[10] Khan O, Kruger J, Trivedi T. Developing passenger mode choice models for Brisbane to reflect observed travel behaviour from the South East Queensland Travel Survey. Melbourne, Australia; 2007.

[11] Black JA. Techniques of land use/transportation planning in Australian cities. Transportation. 1974;3: 255–288. doi: 10.1007/BF00165490

[12] Transport for London. Old Oak Common Strategic Transport Study. London, UK: Transport for London; 2015. Available: https://www.london.gov.uk/sites/default/files/gla_migrate_files_destination/OOC%20Transport%20Study.pdf.

[13] Fox J, Patruni B. Model of Travel in London Phase 3: Mode and destination choice model estimation (Report for Transport for London). Cambridge, UK: RAND Corporation; 2021. doi: 10.7249/RRA1217-1

[14] Inoa IA, Picard N, De Palma A. Effect of an Accessibility Measure in a Model for Choice of Residential Location, Workplace, and Type of Employment. Mathematical Population Studies. 2015;22: 4–36. doi: 10.1080/08898480.2014.925318

[15] Claire P, Dupont-Kieffer A, Palmier P. Potential accessibility to the workplace by public transit and its social distribution in Lille, France: A scenario-based equity appraisal. Transport Policy. 2022;125: 256–266. doi: 10.1016/j.tranpol.2022.06.005

[16] Gunn HF, Tuinenga JG, Aliouche JF, Debrincat L. ANTONIN: A forecasting model for travel demand in the Ile de France, Transport. 26th European Transport Conference. Loughborough, UK: Loughborough University; 1998.

[17] Sullivan J. Analysis of Address-Based Employment and Demographic Data Sources For Travel Modeling and Transportation Planning. Burlington, VT, USA: University of Vermont Transportation Research Center; 2018.

[18] Kimboko A. A direct and behavioral travel demand model for prediction of campground use by urban recreationists. Doctor of Philosophy, Portland State University. 1977. doi: 10.15760/etd.455

[19] National Research Council, editor. Travel demand models: application, limitations and quantitative methods. Washington, DC: TRB; 1980.

[20] Broomhill R, Sharp R. The Changing Male Breadwinner Model in Australia: a New Gender Order? Labour and Industry. 2005;16: 103–127. doi: 10.1080/10301763.2005.10722033

[21] Standing G. The Precariat: From Denizens to Citizens? Polity. 2012;44: 588–608. doi: 10.1057/pol.2012.15

[22] Thévenon O. Drivers of Female Labour Force Participation in the OECD. Paris: OECD Publishing; 2013. doi: 10.1787/5k46cvrgnms6-en

[23] Spreitzer GM, Cameron L, Garrett L. Alternative Work Arrangements: Two Images of the New World of Work. Annu Rev Organ Psychol Organ Behav. 2017;4: 473–499. doi: 10.1146/annurev-orgpsych-032516-113332

[24] Alpaslan-Danisman S. Metaphors of human resources: Colours of collars. In: Raţă G, Arslan H, Runcan P-L, Akdemir A, editors. Interdisciplinary perspectives on social sciences. Newcastle upon Tyne: Cambridge Scholars Publishing; 2014.

[25] Langford DA, Male S. Strategic management in construction. 2nd ed. Malden, MA: Blackwell Science; 2001.

[26] Kuhn KM, Maleki A. Micro-entrepreneurs, Dependent Contractors, and Instaserfs: Understanding Online Labor Platform Workforces. AMP. 2017;31: 183–200. doi: 10.5465/amp.2015.0111

[27] Do Bereitschaft B. “creative” and “non-creative” workers exhibit similar preferences for urban amenities? An exploratory case study of Omaha, Nebraska. Journal of Urbanism: International Research on Placemaking and Urban Sustainability. 2017;10: 198–216. doi: 10.1080/17549175.2016.1223740

[28] Cool Bille T., funky and creative? The creative class and preferences for leisure and culture. International Journal of Cultural Policy. 2010;16: 466–496. doi: 10.1080/10286630903302741

[29] Frenkel A, Bendit E, Kaplan S. The linkage between the lifestyle of knowledge-workers and their intra-metropolitan residential choice: A clustering approach based on self-organizing maps. Computers, Environment and Urban Systems. 2013;39: 151–161. doi: 10.1016/j.compenvurbsys.2012.09.001

[30] Markusen A. Urban Development and the Politics of a Creative Class: Evidence from a Study of Artists. Environ Plan A. 2006;38: 1921–1940. doi: 10.1068/a38179

[31] Clark B, Chatterjee K, Melia S. Changes to commute mode: The role of life events, spatial context and environmental attitude. Transportation Research Part A: Policy and Practice. 2016;89: 89–105. doi: 10.1016/j.tra.2016.05.005

[32] Michigan. State Highway Department. Detroit Metropolitan Area Traffic Study, 1953. Harvard Dataverse; 1955. doi: 10.7910/DVN/NL4F2K

[33] Wilbur Smith and Associates. Brisbane transportation study. Brisbane, Australia: Wilbur Smith and Associates; 1965.

[34] Lennox J. Spatial economic dynamics in transport project appraisal. Economic Modelling. 2023;127: 106464. doi: 10.1016/j.econmod.2023.106464

[35] Collins D, Tisdell C. Gender and Differences in Travel Life Cycles. Journal of Travel Research. 2002;41: 133–143. doi: 10.1177/004728702237413

[36] Crane R. Is There a Quiet Revolution in Women’s Travel? Revisiting the Gender Gap in Commuting. Journal of the American Planning Association. 2007;73: 298–316. doi: 10.1080/01944360708977979

[37] Government Queensland. Queensland Household Travel Survey—2017–20. 2020. Available: https://www.data.qld.gov.au/dataset/queensland-household-travel-survey-series/resource/351cd3dd-939b-43bb-9e88-37cb0cb20c82.

[38] Ben-Akiva ME, Lerman SR. Discrete choice analysis: theory and application to travel demand. Cambridge, Massachusetts London: The MIT Press; 1985.

[39] Koppelman FS, Bhat C. A Self Instructing Course in Mode Choice Modeling: Multinomial and Nested Logit Models. U.S. Department of Transportation and Federal Transit Administration; 2006. Available: https://www.caee.utexas.edu/prof/bhat/courses/lm_draft_060131final-060630.pdf.

[40] Australian Bureau of Statistics. 1220.0—ANZSCO—Australian and New Zealand Standard Classification of Occupations, 2013, Version 1.2. 2013. Available: https://www.abs.gov.au/AUSSTATS/abs@.nsf/allprimarymainfeatures/4AF138F6DB4FFD4BCA2571E200096BAD.

[41] Eick CF, Zeidat N, Zhao Z. Supervised clustering—algorithms and benefits. 16th IEEE International Conference on Tools with Artificial Intelligence. Boca Raton, FL, USA: IEEE Comput. Soc; 2004. pp. 774–776. doi: 10.1109/ICTAI.2004.111

[42] Handl J, Knowles J, Kell DB. Computational cluster validation in post-genomic data analysis. Bioinformatics. 2005;21: 3201–3212. doi: 10.1093/bioinformatics/bti517 15914541

[43] England KVL. Suburban Pink Collar Ghettos: The Spatial Entrapment of Women? Annals of the Association of American Geographers. 1993;83: 225–242. doi: 10.1111/j.1467-8306.1993.tb01933.x

[44] Hensher DA. Market segmentation as a mechanism in allowing for variability of traveller behaviour. Transportation. 1976;5: 257–284. doi: 10.1007/BF00148379

[45] Kubacki K, Dietrich T, Rundle-Thiele S. Segmentation in Social Marketing: Why We Should Do It More Often that We Currently Do. In: Dietrich T, Rundle-Thiele S, Kubacki K, editors. Segmentation in Social Marketing. Singapore: Springer Singapore; 2017. pp. 1–6. doi: 10.1007/978-981-10-1835-0_1

[46] Podani J, Schmera D. On dendrogram-based measures of functional diversity. Oikos. 2006;115: 179–185. doi: 10.1111/j.2006.0030–1299.15048.x

[47] Kuczynski J, Liu Z, Lozupone C, McDonald D, Fierer N, Knight R. Microbial community resemblance methods differ in their ability to detect biologically relevant patterns. Nat Methods. 2010;7: 813–819. doi: 10.1038/nmeth.1499 20818378 PMC2948603

[48] Rousseeuw PJ. Silhouettes: A graphical aid to the interpretation and validation of cluster analysis. Journal of Computational and Applied Mathematics. 1987;20: 53–65. doi: 10.1016/0377-0427(87)90125-7

[49] van der Maaten L, Hinton G. Visualizing High-Dimensional Data Using t-SNE. 2008;9: 2579–2605.

[50] Zapata-Diomedi B, Knibbs LD, Ware RS, Heesch KC, Tainio M, Woodcock J, et al. A shift from motorised travel to active transport: What are the potential health gains for an Australian city? Ebi KL, editor. PLOS ONE. 2017;12: e0184799. doi: 10.1371/journal.pone.0184799 29020093 PMC5636090

[51] Goodman A., Cycling and Driving to Work in the English and Welsh 2011 Census: Trends, Socio-Economic Patterning and Relevance to Travel Behaviour in General. Zhang H, editor. PLoS ONE. 2013;8: e71790. doi: 10.1371/journal.pone.0071790 23990990 PMC3749195

[52] Baxter J, Hand K, Sweed R. Flexible child care and Australian parents’ work and care decision-making (Research Report No. 37). Melbourne, Australia: Australian Institute of Family Studies; 2016. Available: https://aifs.gov.au/sites/default/files/publication-documents/rr37-flexible-child-care.pdf.

