# Supplementary material for: Understanding and including ‘pink-collar’ workers in employment-based travel demand models
Source: PLoS One. 2024 Apr 18;19(4):e0301001. doi: 10.1371/journal.pone.0301001 (PMC11025955; doi:10.1371/journal.pone.0301001)
Supplement: Attachment — Submitted filename: Table of Revisions_v4_proposedfinal.docx [file pone.0301001.s002.docx]

**Table of Revisions**

| **Reviewer 1** | | |
| --- | --- | --- |
| **No.** | **Comment** | **Response** |
| **R1.1** | The paper addresses a very specific topic which is the segmentation of respondents of the Brisbane standard transport surveys. The authors could present their findings in a broader international context.  ….  Only people very familiar with the current practice in that specific city/area could value the findings and the analysis. By explaining the survey and modelling context, the analysis could be better understood and valued. International readers need to highlight the importance of the outputs to other cities/countries.  ….  The results should help not only consultants and transport planners in Brisbane but in other contexts. To that end, the authors should reflect on which findings are general or only valid for local conditions. | Thanks to the reviewer for the helpful critique and suggested improvements.  For this issue, we first:   - Made clearer that this issue is common to transport models in a range of other cities internationally; and, - That similar datasets to those available in Queensland are also available in other nations, even more so where countries have National Household Travel Survey data (Australia being one of the few OECD nations that does not have such a survey).   We’ve made a point of highlighting where the *general* approach and methods are useful in an international context, and being careful to note what is Brisbane-specific. Amongst other changes, this includes the following on p3:  “Cities such as London (12,12,13) and Paris (14–16) in Europe, and many regional models in North America (17–19) have similar blue/white collar market segmentations in their transport models. But more nuanced and differentiated market segmentations are being developed.”  And this on p23:  “…. . The approach also allows each city to go and explore its own datasets and come up with reasonable and justifiable ‘solutions’ for the market segmentation problem, regardless of its peculiar employment market. What may work for New York may be very different to that of Brisbane, and different again in Honolulu. As household travel survey data are increasingly made available freely via open data portals, the methods developed in this paper should help planners, modellers and researchers across a litany of cities improve the accuracy of their models.” |
| **R1.2** | The methods are correctly applied, but the research process needs to be reordered and completed. The statistical analysis been performed correctly, but some steps merit a deeper explanation. Only the results of the analysis are presented. Data sources are mentioned and they are probably available, but they do not mention it. | The most important element of the research process is the PAM clustering is explained exhaustively. We do not know what more the reviewer might be wanting to further explain this work. The data is explained, each step in the process is explained, what more could one want? All the key elements are already included in the paper.  That said, we did see that the step of taking the clusters, and then identifying their travel behaviours, was not outlined earlier in the methods, especially which variables were going to be calculated and how. This was included in Section … Page XX, Line XX, which now says:   - *“Once market segments were identified, the travel behaviour of these groups was then explored using* Python *with the same SEQTS dataset.* *The variables calculated included: mode shares; and, trip distances by mode, with the latter represented as violin plots for ease of interpretation.”*   We used an open portal dataset. The following has been added to make clear where the dataset is available.   - *“This survey is based on the* South East Queensland Travel Survey 2017-2020 *(SEQTS), made publicly available in an open data portal by the Queensland Department of Transport and Main Roads (DTMR) (40).“*   *…*  *“More information on the data collection process is available from DTMR (40).*  These changes better structure and order the narrative, provide key information on the methods previously omitted, and fix the issue of the data sources. |
| **R1.3** | The paper refers several times along the paper to the initial motivation related to updating the old division of trips between blue and white collar workers. Once at the beginning is enough. | Fixed.  We have removed three references to this in later parts of the revised paper, including on page 6. |
| **R1.4** | Another general comment is about the type of trips considered in the analysis; the segmentation analysis referred only to commuting trips, but it is not clearly said, that only those trips are selected. The issue is not only to survey workers but for their trip to work. | Fixed.  Lines 380-381 now read: “… shows commuting behaviours of the three clusters based on respondents’ journey-to-work trips only”. |
| **R1.5** | The introduction makes a very detailed reference to ANZSCO, which is explained later in section 2.2. The introduction could only refer to the problem and then describe it. | Fixed.  The relevant text is relocated to the methodology section. |
| **R1.6** | Chapter 3 includes the methodology. However, it needs to start to describe and define the characteristics of the SEQTS and BSTM. | Fixed.  The description of the SEQTS and BSTM is now moved up with a short statement of the relevant characteristics of the dataset and the model, and a statement of where to go for further detail on the data, as per above. |
| **R1.7** | Clarify which means active transport. If walking and cycling or something else, like multimodal trips with some stages not motorized. | Fixed.  A footnote is added to Table 3 to clarify that:  “Note: Active transport includes journeys made by walking only, or by cycling, but does not include public transport trips where these modes were used for the first or last mile.” |
| **R1.8** | The clusters are based on a number of variables, including the trip length for each mode. It would be nice to comment on the text which is the average/median distance for all. I understand that trip distance refers to the Origin-Destination route. It surprises me that PT trips have longer itineraries than private cars. Normally people use cars because they live/work in more distant places. If not, then the household economic level should be included as a variable. Which means CBD? Clarify. | We see what the reviewer is getting at here. The key part of this comment is the somewhat unusual commute lengths by public transport. This is true of the SEQ region, but not of Brisbane City, per se. We thought how to resolve this and the following explanation is added as a footnote to Table 3 to include a lengthy description, whilst trying to limit page length overall.  “The clusters are based on a number of variables, including the median trip length for private motorised vehicles, public transport and active transport. Public transport trips being longer than the private car trips was unexpected. There are reasons for this outcome. Brisbane’s express busway and rail networks carry many suburban commuters into the centre; there are many thousands of inter-city commute trips made daily, including between the Gold Coast, Ipswich, the Sunshine Coast and Brisbane; there are almost no cross-suburban public transport routes, requiring transfers in Brisbane’s CBD; and, the city’s road networks tend to offer more direct routes between O-D pairs, and lesser distances, than the rail corridors.”  CBD is now expanded to “Central Business District”. |
| **Reviewer 2** | | |
| **No.** | **Comment** | **Response** |
| **R2.1** | This study can make a useful contribution: I can see how three clusters, one of which largely identifies pink collar  workers, are an improvement over two clusters, largely representing blue and white collar workers, respectively. | Thank you. |
| **R2.2** | However, much of the paper’s core argumentation is unclear and/or illogical.  In particular, the claim that older segmentations were “deductive”, based on modelers’ (presumptively biased) hunches  about appropriate segmentation variables, while the present one is “inductive”, “let[ting the] data speak for itself” (line  62), seems quite overdrawn to me. Biases are not excluded from the putatively data-driven approach of the present  study either. First of all (and through no fault of the authors), previous biases and hunches – about what is appropriate  and important to measure – have determined what variables are even *available* to the cluster analysis. Second, the  *present* analysts’ hunches about what variables are relevant to identifying segments that “place different importance  on different aspects of service” (line 241) are governing *this* analysis as much as *prior* analysts’ hunches governed  *theirs*. For example, the present cluster analysis includes a number of variables, but apparently does not include race,  sexual orientation, income, presence of children, ages of children, number of elders, number of wage-earners, nor transit  pass ownership, as well as spatial and attitudinal characteristics (as mentioned by the authors). Some those variables  may not be available in the dataset (see the “first of all” point above), but some presumably are available, and were  consciously or unconsciously rejected by the authors – on their hunch – as being insufficiently relevant.  And by the way, if you want to be even *more* data-driven, why not use latent class mode choice and other travel  behavior models, to identify the *segments that best differentiate the segment-specific coefficients* of the outcome  model? As it is, the authors are still performing a deterministic segmentation in an entirely separate step from the travel  behavior model to which they see the clusters eventually being applied, with no guarantee that this is the *best*  segmentation for the purposes of identifying segments that “place different importance on different aspects of service”.  I recognize that a latent class choice model is a different paper than this one, and I’m not insisting that this one be  replaced with a latent class choice model. I am simply saying that the authors need to be more objective and logically  consistent about their characterizations of the alternative approaches that have been taken. Best to suggest that  analysts in each era probably do the best job they can of objectively considering what’s important, but that no analysts,  including the present ones, are bias-free, and it is quite possible (even likely) that future analysts will criticize the  present study for omitting important variables that society later recognizes as critical – in the same way that the present  study is criticizing previous studies for doing the same.  In short, please remove discussion of what I see as a false dichotomy between deductive and inductive approaches, and  do not claim (line 181) that “Inductive approaches are better suited for this type of analysis as they remove most  potential researcher bias  lines 408-410, “In applied terms, the results provide strong empirical evidence that the conventional blue/white collar  segmentation strategy for transport models fails to sufficiently represent female workers’ travel behaviour appropriately,  at least in Australia”: Not really. If included at all, the statement needs to be softened to something like, “The results  suggest that the conventional blue/white collar segmentation strategy for transport models may fail to sufficiently  represent female workers’ travel behaviour appropriately, at least in Australia. However, this conjecture should be tested  by comparing the predictive ability, by sex, of models using a blue/ white collar segmentation to that of models using the  blue/white/pink collar segments identified in this paper.” | We have been praised by some members of the Australian modelling community and at the same time criticised by other more defensive members in industry along similar lines (i.e. “there are other biases in the data”; “what about your own biases?”; “might some other approach go further?”). We’ve taken this on board. But we do stand by our general claim that this white-/blue-collar market segmentation is one more obvious bias that, handed down from generation-to-generation, really should be critiqued and rectified.  That said, we’ve made a large number of conciliatory changes in response to the reviewer’s comments.  Firstly, we have reduced the ‘admonitory tone’ that the reviewer appears to have taken some offence to.  Second, we added some key self-reflection directly in line with the reviewer’s critique.  Third, just note that the reviewer was right that we removed a number of variables for co-linearity, as discussed. But otherwise we used all the known travel behaviour variables available in the SEQTS dataset. In other words we were not bringing our own biases to the independent variable selection problem; their critique on that matter is unfounded).  Changes made include:   - Stated the criteria for variable selection “*For the choice of variables to form the basis of clustering, it is based on data availability, guidance from prior works (43–45), and also elimination of variables that are too similar (to avoid co-linearity).*” - We make clear on page 10, line 258, that:   *“Although the cluster analysis is inductive, it omits variables that also could influence travel behaviour. The SEQTS itself does not have any attitudinal data, such as environmental attitudes or attachment to particular modes like driving. These are major limitations, which afflict many other HTS datasets.”*   - The Discussion (page 19, line 411) now includes   the following statements:  *“Our analysis is just one modest step in identifying potential improvements. What we have found should be tested for its predictability. That is, testing how a refined BSTM performs using the blue-/white-collar segmentation, compared to the blue-/white-/pink-segmentation. Then there are other data-driven approaches that could take this work further. Latent class mode choice studies could provide a richer picture and additional improvements.”*  *…*  And a new ending to the paper (line 450):  *“Methodological and cultural change will continue in the future. Transport researchers may look back at this paper and admonish it for omitting a litany of new variables, and for its conscious and unconscious decisions. That is to be both expected, and encouraged.”* |
| **R2.3** | But I’m also uneasy about the multiple references to it being *women’s* travel behavior that is being overlooked or  misrepresented (e.g. lines 39, 54-55, 164-166, 410). The white-collar segment of this study is 64% women/36% men,  so in this important segment, women and men are still being smushed together – as are people with different numbers  of children, seniors, adults, workers, and drivers in the household, etc. If it’s truly *women’s* travel behavior that needs  to be clearly distinguished, then a deterministic split between women and men should be an essential component of the  segmentation process, regardless of what other variables might also be considered. Instead though, I gather that it may  be *pink-collar workers’* travel behavior (most of whom are women, but far from all women are pink-collar) that is  being subsumed under cruder segmentation systems. I agree that we don’t want to do that, but let’s call the problem  what it is, and be precise about what the study is actually doing. The paper issues a caveat along these lines at lines 403  – 406, but more or less ignores that caveat for the preceding 95% of the paper. How *should* someone “tak[e] these  results through and operationalis[e] them in mode choice models”? It seems like a pretty important question for the  paper to answer, in view of its admonitory tone throughout. | Importantly, we wish to note that the white-collar segment produced AFTER our methods are applied was 64% women. And as shown at k = 8, and in our discussion of the results, there are a set of smaller sub-groups (male-dominated and female-dominated) that ‘merge’ into this cluster with aggregation.  We have tried to make clear – throughout – that the ‘pink-collar’ workforce is female-dominant, not just women.  We have also noted that the inductive approach would tell us what mattered re variables, not us using a deterministic split straight up. Despite this at k = 8 the data was very, very heavily split by gender, using our approach.  Again, in a conciliatory tone, we have made a number of key changes in line with this critique. These include the key message that those who used such a deterministic split were certainly looking in the right place! The changes include the follow additions/changes to the Discussion:  Page 19:  *“Firstly, the results confirm that those who deductively identified and included pink-collar market segments in urban transport model elsewhere were on the right track.”*  *…*  *“There are limitations and one must be cautious in interpreting the results. Female-dominated or male-dominated clusters, and their travel behaviour, should not be conflated with the commuting behaviour of men or women, per se.”* |
| **R2.4** | Also, the paper needs a thorough edit for grammar, punctuation, and typos. Time doesn’t permit an exhaustive list, but  one example is at line 142, “the forebear of that work”. “Forebear” means “ancestor”, whereas the context requires it to  say “descendent”. | The paper was given another proof edit. Over sixty tidy-ups and minor changes were made to the text, fixing a series of textual and contextual errors. |
| **R2.5** | In the title, “sublimation” (and, at line 165, “sublimated”) is not really the right word, unless there is some Australian  distinction that is unknown to this native American English speaker (and therefore to a large share of this paper’s  potential audience). I recommend “subsumation”, consistent with the paper’s use of “subsumed” at line 54. | Fixed throughout.  A much clearer title is now provided that also removes the term entirely. |
| **R2.6** | line 20, “Conventional transport models tend to segment commuters as either blue or white collar workers”: I would say,  “often” (or, really, “sometimes”), since that particular segmentation is not at all common in my experience. It’s not even  clear if it’s any longer common in Australia – the paper (line 53-54) refers to “key Australian transport models of the  past”, but what about the present? Please confirm that references 6-10 provide “lots” of examples of the *current* use  of that segmentation. OK, lines 141-142 give one current example – are there others? Enough to justify use of the  phrase “tend to”? Even if so, the contention should still be qualified with “*Australian* transport models”. | Note the changes made on this matter re international city models, above, (Reviewer’s 1’s concerns). We’ve added 5-6 references to support our claims. |
| **R2.7** | line 57: Although not all new things are novel, all novel things are, by definition, new, so it suffices to say “novel typology”, rather than “new and novel typology”. | This is now amended – ‘new’ removed |
| **R2.8** | line 214: I was a little surprised that the SEQTS data represented an average per-person trip rate of 2.8 trips per day. In  the US it is 3.4 trips per person per day (https://nhts.ornl.gov/assets/2017_nhts_summary_travel_trends.pdf), and I  wouldn’t have thought that the Southeast Queensland region would be so different. | We were going to clarify this by writing:  *“The SEQ trip rate of 2.8 trips per day is less than the US national average of 3.4 trips per day. However, there are significant differences between the SEQTS survey methods and those of the USA’s NHTS. Care should be taken in comparing these trip rates.”*  BUT, then the latest US NHTS data came out, using their new methods, and guess what happened? The US trip rate was suddenly a lot, lot lower. It was the talk of the TRB conference in January, and I’ve seen a lot of chatter online about it (including some comment about methods, interpretations, whether it was just Covid-19, etc.).  We’d prefer to just leave this be and NOT action this request please. |
| **R2.9** | lines 270-275: This was rather confusingly written. I would like to rewrite it to say, “Most simply, Gower distance is  computed as the average of partial dissimilarities across individuals, in which each numeric-valued feature is  standardized, and the distance is calculated as the average of all feature-specific distances. However, if variables are of  mixed (qualitative as well as numeric) types, partial dissimilarity is calculated differently. For numeric features, it is  computed as the ratio between absolute difference of observations Xi and Xj and maximum range observed from all  individuals (thus scaling all dissimilarities to lie between 0 for identical, and 1 for maximally dissimilar):” If this  characterization is correct, it would be much clearer to me for it to be presented this way. | We thank the reviewer for this comment, and the suggested text is used as a replacement. |
| **R2.10** | lines 305-307, “each cluster is dominated by certain industries and occupations sharing similar commute mode  preferences and similar work trip distances”: For a given cluster, it is not clear how similar mode preferences and  commute distances are within industry and occupation, since each cluster comprises an undifferentiated bundle of  differing shares of industries and occupations. I suggest just focusing on distinct differences across clusters. | Removed. |
| **R2.11** | Table 4: The white collar and pink collar column heads appear to be reversed, based on Figure 3 and statements in the text. I.e. the information in the second column pertains to the pink collar cluster and the third column describes the white collar cluster.  And regarding the variables from CBD down, are we talking about commute trips specifically? Please clarify; also in Table 3. | Fixed.  Good spot by the reviewer. Thanks.  Fixed. See response to same issue for Reviewer 1. |
| **R2.12** | Figure 3: Many readers (including this one) will not have the foggiest idea what the “t-Distributed Stochastic Neighbour  Embedding(t-SNE) algorithm” is, and accordingly have no way of interpreting.  Figure 3. Please at least explain what the  two axes represent, and preferably also give a brief explanation of what the t-SNE algorithm is and does. | This is now provided with added explanation:  “*The t-Distributed Stochastic Neighbour Embedding (t-SNE) method is a specialised technique for reducing dimensionality, making it especially effective for visualising high-dimensional data within a low-dimensional space (e.g., two- or three-dimensional) while preserving local structure (51). Using t-SNE, the data can be visualised into a two-dimensional plot (Fig 3) showing the clusters of the blue, white and pink collar k = 3 solution as presented in Table 4. It should be noted that this is visualisation technique to tell how far apart the clusters are in a low-dimensional, they not meant for quantitative interpretation.”*  It is a common practice not to label the axes in such visualisation technique (see the referenced paper). |
| **Reviewer 3** | | |
| **No.** | **Comment** | **Response** |
| **E1.1** | Although the paper's aim is challenging, it must be reordered and completed. The analysis has been performed correctly, but some parts need a deeper explanation. | This is very similar to issues raised by Reviewer 1. A series of changes were made in line with these comments. See above. |
|  | The cluster analysis is based only on a few variables, but other relevant variables are not referred to in the study, such as race, age, etc. On the other hand, the paper's core argumentation is unclear, and the claim of the present segmentation is "inductive," whereas the older ones were "deductive," which is challenged by several inconsistencies throughout the manuscript. | These issues are now tidied up significantly, as noted in the extensive changes made to respond to Reviewer 2. See comment R2.2, above. |
|  | In addition, the study suggests that conventional blue/white collar segmentation strategy for transport models may fail to represent female workers' travel behavior in Australia adequately and that a deterministic split between women and men should be an essential component of the segmentation process, but these claims must be substantiated with more evidence. The empirical analysis lacks a more comprehensive justification. | With great respect we are not saying “*a deterministic split between women and men should be an essential component*”. That’s a bit of over-reach. We are suggesting that the traditional blue- and white-collar market segmentations aren’t representing well a large sub-set of commuters. And the data analysis on the Brisbane data suggests it is this pink-collar market segment that should be included. We’ve provided that evidence.  That said, we have taken the following actions throughout the paper:   - We’ve toned down some of the language and improved the narrative to not be as admonishing; - We’ve improved the justification for the approach, and highlighted its limitations; - We’ve been more self-reflective, acknowledging we are still bringing our own conscious and unconscious biases to this analysis. |
|  | The database must be provided, or a link where it can be accessed. | We used an open portal dataset. The following has been added to make clear where the dataset is available.  *“This survey is based on the South East Queensland Travel Survey 2017-2020 (SEQTS), which is made publicly available in an open data portal by the Department of Transport and Main Roads (DTMR) (42).“*  *(same as R1.4)* |
|  | Similarly, all subjective opinions and qualifications must be supported with documented evidence, preferably using a summarizing list or table. | The subjective opinions and admonitory wordings are now toned now and written in a more objective tone. |
|  | Many references are more than ten years old. | With great respect, citing the older, foundational studies on things like travel time budgets is a sensible thing to do here, and is an approach regularly used by many top-1% professors in this field-of-research. We also note that older papers are often better at clarifying issues in a mature and well-established, but not-too-fast-moving, field-of-research. Travel behaviour studies and transport modelling fit this definition.  Nevertheless, some newer papers are now added to provide better relevancy. |
|  | since the manuscript has already been presented at a congress, a statement must be added to mention the paper's character as the updated version of that submission. | This is now added. |
|  | Perform a comprehensive revision for typography errors and punctuation. | Undertaken, as noted above. |
|  | Review the manuscript's structure to align the title with its content and aims. | The title is changed and the paper is now better aligned, as well as improvements made to the structure of the document, in line with Reviewer 1’s request. |
|  | State the advantages and limitations of the methodology  …  The usage of the clustering approach needs a more detailed explanation to mention the method's advantages and drawbacks. | A litany of changes were made to better explain the clustering approach, and such issues as the t-SNE algorithm (see above).  We’ve also added the following in page 10 to discuss the key advantages/disadvantages of the PAM approach:  *“Unsupervised methods have an advantage over traditional clustering methods (such as hierarchical) as it do not require prior knowledge and hence less subjective. Also, compared to the more commonly used k-means clustering algorithm, PAM is more intuitive and robust to noise and outliers in the underlying data, due to the properties of distances being used. In addition, PAM is capable of analysing mixed-type data, where numeric, nominal, or ordinal features coexist. The main disadvantage of K-medoid algorithms (such as PAM) is that they are not suitable for clustering non-spherical (arbitrary shaped) groups of objects. In our case, this limitation does not apply as the clustering formed spherical shapes (Fig 3).”* |
|  | Provide access to the database | We used an open portal dataset. The following has been added to make clear where the dataset is available.  *“This survey is based on the South East Queensland Travel Survey 2017-2020 (SEQTS), which is made publicly available in an open data portal by the Department of Transport and Main Roads (DTMR) (42).“ (same as R1.4)* |
|  | Clarify and explain all technical terms and define their meaning when necessary.  …  There must be a careful usage of technical and methodological terms to avoid misleading conceptualizations. In particular, a thoughtful revision of the conceptual meanings, such as sublimation, CBD, etc., must be carried out. | See responses to R1.4, R1.6, and R1.7, above. |
|  | Add a conclusion  …  The manuscript lacks a section dealing with the main conclusions of the study. As a result, there are no comments on the study's policy implications and a proposal for future research. | See additions including the new concluding section, which includes paragraphs that respond to comment R2.2, above. |
|  | The bibliography must be updated. | Fixed. |
